# Whole-body magnetic resonance imaging for the diagnosis of metastasis in children and adolescents: a systematic review and meta-analysis

**DOI:** 10.1590/0100-3984.2020.0183

**Published:** 2021

**Authors:** Simone Gianella Valduga, Gabriele Carra Forte, Ricardo Pessini Paganin, Diego Gomez Abreu, Tássia Machado Medeiros, Klaus Irion, Bruno Hochhegger, Rita Mattiello

**Affiliations:** 1 Pontifícia Universidade Católica do Rio Grande do Sul (PUCRS), Porto Alegre, RS, Brazil.; 2 Universidad Industrial de Santander (UIS), Bucaramanga, Santander, Colombia.; 3 Thoracic Imaging DIIRM, Manchester University and Manchester University NHS Foundation Trust Department of Radiology, Manchester, UK.

**Keywords:** Whole body imaging/methods, Magnetic resonance imaging/methods, Neoplasms/diagnostic imaging, Meta-analysis, Pediatrics, Imagem corporal total/métodos, Ressonância magnética/métodos, Neoplasias/diagnóstico por imagem, Metanálise, Pediatria

## Abstract

Whole-body magnetic resonance imaging (WB-MRI) is a noninvasive imaging method that can be used to diagnose and stage tumors, as well as to assess therapeutic responses in oncology. The objective of this meta-analysis was to evaluate the accuracy of WB-MRI for the diagnosis of metastases in pediatric patients. The following electronic databases were searched: Medline, Embase, Cochrane Central Register of Controlled Trials, Scientific Electronic Library Online, Latin-American and Caribbean Health Sciences Literature, Cumulative Index to Nursing and Allied Health Literature, Web of Science, and ClinicalTrials.gov. All of the selected studies included children and adolescents with histopathological confirmation of a primary tumor. Collectively, the studies included 118 patients ranging in age from 7 months to 19 years. The pooled sensitivity and specificity of WB-MRI were, respectively, 0.964 (95% CI: 0.944-0.978; *I*^2^ = 0%) and 0.902 (95% CI: 0.882-0.919; *I*^2^ = 98.4%), with an area under the curve (AUC) of 0.991. We found that WB-MRI had good accuracy for the diagnosis of metastases in pediatric patients and could therefore provide an alternative to complete the staging of tumors in such patients, being a safer option because it does not involve the use of ionizing radiation.

## Abstract (pt)

A ressonância magnética de corpo inteiro (WB-MRI) é um método de imagem não invasivo que pode ser usado para diagnosticar, estadiar e avaliar a resposta terapêutica em oncologia. O objetivo desta meta-análise foi avaliar a precisão do diagnóstico de WB- MRI no diagnóstico de metástases em crianças. Foram pesquisadas as seguintes bastes de dados: Medline, Embase, Cochrane Central Register of Controlled Trials, Scientific Electronic Library Online, Latin-American and Caribbean Health Sciences Literature, Cumulative Index to Nursing and Allied Health Literature, Web of Science, and ClinicalTrials.gov. Todos os estudos incluíram crianças e adolescentes com prova histopatológica de um tumor original. Os estudos selecionados incluíram 118 pacientes com idade variando de 7 meses a 19 anos. A sensibilidade e especificidade combinadas de WB-MRI foram, respectivamente, 0,964 (IC 95%: 0,944-0,978; *I*^2^ = 0%) e 0,902 (IC 95%: 0,882-0,919; *I*^2^ = 98,4%), com AUC de 0,991. A WB-MRI tem uma boa precisão para o diagnóstico de metástases em pediatria e pode potencialmente fornecer um método alternativo não ionizante mais seguro para completar o estadiamento da doença maligna em crianças.

## INTRODUCTION

Cancer has long been considered a major risk factor for death in the pediatric population^(1)^. However, when cancer is diagnosed at an early stage, the chances of cure can be high^(1,2)^. Various imaging methods have been used in order to assess the extent of local and distant disease^(3)^. Ideally, imaging examinations should be rapid, should provide high quality diagnostic information, and should be safe^(2,4,5)^.

Imaging methods that use ionizing radiation, such as X-ray, computed tomography (CT), bone scintigraphy, and positron emission tomography/CT (PET/CT) are still routinely employed^(2,4,5)^. However, exposure to ionizing radiation is a major consideration in pediatric patients with cancer. Recent improvements in magnetic resonance imaging (MRI) hardware and software have dramatically reduced scanning times, thus making MRI more appropriate for use in children^(2,4,6)^.

Whole-body MRI (WB-MRI) is a noninvasive imaging method that can be used to diagnose and stage tumors, as well as to assess therapeutic responses in oncology^(7,8)^. In addition to providing coverage of the entire body, WB-MRI produces images with excellent soft tissue contrast and spatial resolution. More importantly, it does not expose patients to radiation, which makes it the ideal method for assessing the primary lesion and systemic spread in pediatric patients. The effectiveness of WB-MRI has been tested in staging patients with known malignancies, as well as in screening patients with genetic predisposition for malignancy^(7,8)^. One recent systematic review assessed the sensitivity of WB-MRI for the detection solely of skeletal metastases in children with primary solid tumors and found promising results^(9)^.

The superior soft tissue contrast and more detailed depiction of bony structures are recognized as advantages of WB-MRI over PET/CT^(2,10,11)^. Although the use of WB-MRI in adults as an alternative to PET/CT is well established, its use for the assessment of metastases is not routine, especially in children and adolescents^(2)^.

The objective of this systematic review and meta-analysis was to evaluate the diagnostic accuracy of WB-MRI for metastatic disease in children and adolescents.

## MATERIALS AND METHODS

### Protocol and registration

This systematic review and meta-analysis was registered with the International Prospective Register of Systematic Reviews (Registration no. CRD42018114271). The protocol was developed in accordance with the Cochrane Collaboration recommendations^(12)^.

### Eligibility criteria

We included studies that evaluated metastatic lesions in pediatric patients using WB-MRI and PET/CT. Editorials and review articles were excluded, as were animal studies and studies that did not include an index test. We imposed no restrictions regarding the language or date of publication.

### Study selection

The following electronic databases were searched: Medline (via PubMed), Embase, Cochrane Central Register of Controlled Trials, Scientific Electronic Library Online, Latin American and Caribbean Health Sciences Literature (via the Brazilian Regional Library of Medicine), Cumulative Index to Nursing and Allied Health Literature, Web of Science, and Clinical Trials.gov. The Medline search strategy was adopted for all of the databases. Additional references were identified through manual searches of the bibliographies of the full-text papers retrieved. In addition, we reviewed the gray literature to identify papers authored by leading experts in the field, as well as manually searching the reference lists of other recent systematic reviews.

### Data collection

Two of the authors, working independently, reviewed the abstracts and titles of the eligible studies. The full texts of the potentially relevant articles were then evaluated. Disagreements were resolved through discussion with a third investigator.

The following data were extracted by using a standardized instrument: first author; year of publication; study design; study population (number, age, and sex of the patients); rates of mortality and adverse events; dose of fluorodeoxyglucose (FDG) employed; time from FDG administration to scanning; comparator imaging test; patient preparation; test interpretation; interval between index and comparator tests (≤ or > three months); assessors (number, expertise, experience, consensus procedures, and learning effect data); and the number of patients whose results were confirmed by each type of reference.

### Risk of bias (quality) assessment

We screened the selected articles for risk of bias. Disagreements were resolved by consensus. Where additional information required review, we reassessed the study after that information had been obtained from the authors of the article in question. The was used in order to assess the methodological quality of the included studies, we employed the Quality Assessment of Diagnostic Accuracy Studies 2 tool^(13)^.

### Strategy for data synthesis

#### Timing and effect measures

A meta-analysis was performed according to the recommendations of the Cochrane Handbook for Systematic Reviews of Diagnostic Test Accuracy^(12)^. The software package Review Manager 5.3 and R were used to conduct meta-analysis. For each study, 2 × 2 contingency tables consisting of true-positives, false-positives, false-negatives, and true-negatives for metastasis were extracted or reconstructed. Sensitivities, specificities, positive predictive values, negative predictive values, and diagnostic odds ratios with 95% confidence intervals (CIs) were recalculated. Diagnostic criteria and cutoff values for metastasis were also extracted. A sensitivity analysis was performed to assess the robustness of analyses by excluding studies from the overall analysis of heterogeneity. The heterogeneity of the articles was further assessed by visually inspecting forest plots and by performing chi-square tests (values of *p* < 0.1 indicating heterogeneity). We used the *I*^*2*^ statistic to quantify inconsistencies among the studies, an *I*^*2*^ > 50% being indicative of substantial heterogeneity.

## RESULTS

**Figure 1.**
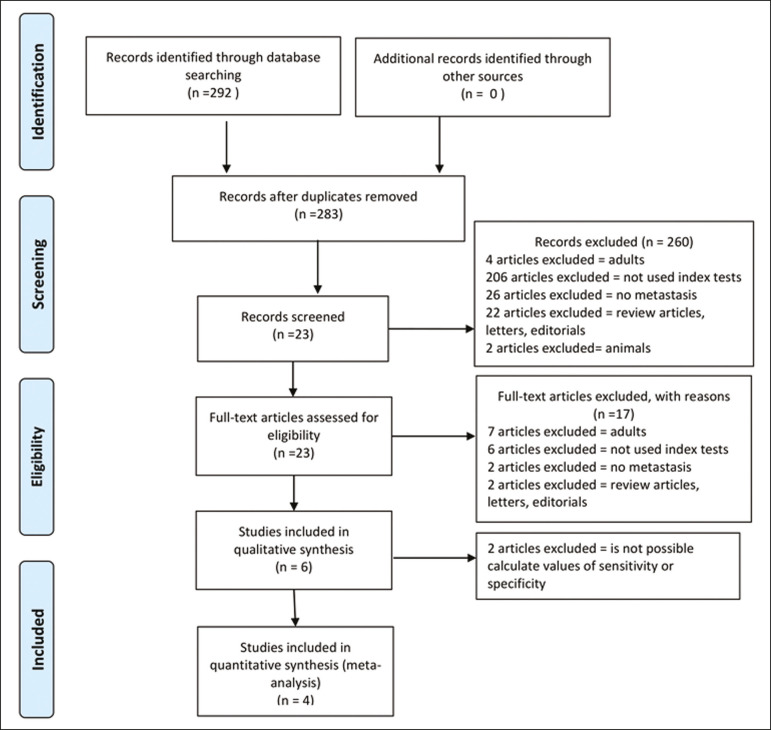
Flow chart of the study selection process.

Figure 1 provides an overview of the literature search and study selection process. After duplicates had been removed, there were 283 eligible studies. During the evaluation of the titles and abstracts, we excluded 260 studies, for the following reasons: adult patients were included (n = 4); no index test was included (n = 206); there was no search for metastases (n = 26); the article type was a review article, letter, or editorial (n = 22); or it was an animal study (n = 2). The remaining 23 articles were then read in full. Of those, another 17 articles were excluded: for including adult patients (n = 7); for not including an index test (n = 6); for not searching for metastasis (n = 2); or for being a review article, letter, or editorial (n = 2). The six remaining articles were included in the systematic review, although two were excluded from the meta-analysis: one because the index study was PET only, rather than PET/CT; and one because the specificity and sensitivity values could not be calculated. Hence, only four articles were included in the meta-analysis.

### Characteristics of the studies included in the systematic review and meta-analysis

Table 1 shows the characteristics of the six studies included in the systematic review. The studies included a collective total of 118 patients, ranging in age from seven months to 19 years. All of the studies included children and adolescents with histopathological confirmation of the primary tumor. The mean sample size was 23 (range, 13-31). Of the 118 patients, 45 (66%) were male. Patients in whom there was incongruence between the findings of the two imaging modalities were further evaluated on the basis of the clinical outcomes^(14)^, focal imaging findings^(14,16)^, findings on follow-up evaluation, and expert panel review^(15)^.

**Table 1:** General characteristics of the selected articles

Authors	Study design	Population	Inclusion criterion	Index test	Blind	WB-MRI parameters	PET/CT parameters	Confirmed (n)
Kumar et al.**^(14)^**	Cross-sectional	N = 26; 62% males; age range, 0.58-18 years	Small-cell neoplasm	Yes	Yes	1.5 T, STIR, T1W	5,254 MBq/kg, 45 min*	26
Punwani et al.**^(15)^**	Cross-sectional	N = 29; 62% males; age range, 5-20 years	Lymphoma (26 Hodgkin and 3 non-Hodgkin)	Yes	Yes	1.5 T, STIR, RARE	5,254 MBq/kg, 60 min*	29
Ishiguchi et al.**^(16)^**	Cross-sectional	N = 13; 54% males; mean age, 2.9 ± 2.0 years	Neuroblastoma	Yes	Yes	1.5 T, STIR, DWIBS	3.7 MBq/kg, 50 min*	13
Daldrup-Link et al.**^(8)^**	Cross-sectional	N = 39; 69% males; age range, 2-19 years	Primary tumor with potential to metastasize to bone	Yes	Yes	1.5 T, STIR, T1W	3.7 MBq/kg, 60 min*	39
Littooij et al.**^(17)^**	Cross-sectional	N = 33; 61% males; age range, 6-21 years	Lymphoma	Yes	Yes	1.5 T, STIR, T1W, DWIBS	5.18-7.4 MBq/kg, 60 min*	33
Latifoltojar et al.**^(18)^**	Cross-sectional	N = 50; 64% males; age range, 5-20 years	Hodgkin lymphoma	Yes	Yes	1.5 T, STIR, T1W FLASH, DWIBS	3.7 MBq/kg, 60 min*	50

T1W, Tl-weighted; RARE, rapid acquisition with relaxation enhancement; FLASH, fast low-angle shot.

* Administration time.

Five of the articles evaluated some form of WB-MRI in comparison with FDG-PET/CT^(8)^. Whole body diffusion-weighted imaging with background body signal suppression (DWIBS) was employed in three studies^(16-18)^, fast spin-echo short-tau inversion-recovery (STIR) sequences were used in two studies^(14,16)^ and T1-weighted spin-echo sequences were used in one study^(8)^. The maximum interval between the two examinations was 20 days. Metastases to bone and lymph nodes were found^(14-16)^. Three studies also investigated the diagnostic performance of the examinations for extranodal disease^(15,17,18)^. All WB-MRI studies were interpreted by two radiologists, whereas all PET/CT studies were interpreted by two nuclear medicine specialists.

### Findings

**Figure 2.**
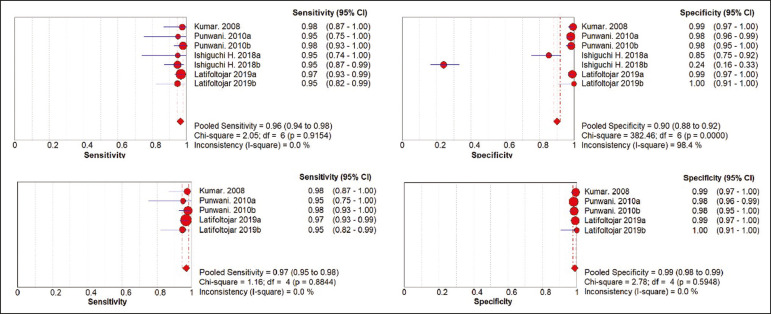
Forest plots of the sensitivity and specificity of WB-MRI.

The summary estimates obtained from the analysis of WB-MRI were a sensitivity of 0.964 (95% CI: 0.944-0.978; *I^2^* = 0%) and a specificity of 0.902 (95% CI: 0.882-0.919; *I^2^* = 98.4%). Although the heterogeneity of the sensitivity values was low (*I^2^* = 0%), that of the specificity values was high (*I^2^* = 98.4%), with an area under the curve of 0.991 (Figure 2). The study conducted by Ishiguchi et al.^(16)^ was excluded from the heterogeneity analysis because it was found to have a high risk of bias. Thereafter, the heterogeneity of both measures decreased to 0%. In that scenario, WB-MRI identified metastasis at 778 sites in 55 patients.

The summary estimates obtained from the analysis of FDG-PET/CT were a sensitivity of 0.916 (95% CI: 0.876-0.947) and specificity of 0.958 (95% CI: 0.942-0.971). The heterogeneity of the sensitivity and specificity values was high (*I^2^* = 85.3% and *I^2^* = 95.4%, respectively). In the sensitivity analysis, performed in order to assess the robustness of our data, excluding studies from the overall analysis of the risk of bias did not decrease the heterogeneity of the sensitivity and specificity values.

### Methodological quality assessment

For all of the studies evaluated, the risk of bias was classified as low, because they met the criteria for one or more of the 14 high risk of bias domains. The individual assessments for each study are shown in Table 2.

**Table 2:** Test interpretation of the selected articles

Authors	Test interpretation
Kumar et al.**^(14)^**	For each patient, metastases were recorded according to body region. The body was divided into eight regions for the purpose of localiza­tion of metastases. On turbo STIR images, skeletal metastases were defined as focal or diffuse hyperintensity of marrow, greater than or equal to the signal intensity of cerebrospinal fluid. On T1-weighted images, marrow metastases were defined as areas of hypointensity, less than or equal to the signal intensity of skeletal muscle. With systemic sclerosis or on FDG-PET, metastatic disease was defined as a focal area of increased radionuclide uptake relative to adjacent and/or contralateral normal tissue.
Punwani et al.**^(15)^**	MRI: The body was divided into 11 nodal areas by standard anatomic definitions. Disease positivity was defined as a mass with a short­axis dimension greater than 1 cm. PET/TC: Disease positivity was defined as the presence of lymph nodes with focal FDG uptake greater than that of the background.
Ishiguchi et al.**^(16)^**	The presence of lymph node metastasis was assessed in eight regions. Bone metastasis was investigated in 17 bone segments. On whole-body DWIBS, the signal intensity of skeletal muscles was used as the reference standard for the judgment of positive results. On ^18^F-FDG PET/CT, ^123^I-metaiodobenzylguanidine scintigraphy/single-photon-emission CT/CT, and bone scintigraphy/single-photon- emission CT, the loci where uptake was visibly higher than the activity of adjacent areas were considered uptake-positive. On CT, charac­teristic massive lesions corresponding to the sites of lymph nodes were defined as metastasis-positive findings, as were focal or diffuse skeletal lesions with or without deformity of cortical bone.
Daldrup-Link et al.**^(8)^**	For all imaging modalities, lesion number and location were determined. On T1-weighted spin-echo images, a metastatic bone or bone marrow lesion was defined as focal or diffuse hypointense bone marrow signal intensity relative to adjacent normal bone marrow. In patients over 10 years of age, neoplastic marrow was defined as that with a signal that was hypointense or isointense in relation to the adjacent muscle tissue. FDG-PET: a metastatic bone lesion was defined as a focal area of increased radionuclide uptake relative to the adjacent and contralateral normal tissue.
Littooij et al.**^(17)^**	Nodal regions included cervical (including supraclavicular nodal site), axillary, infraclavicular, mediastinal, hilar, para-aortic, mesenteric, pelvic and inguinal lymph node regions. Lymph nodes were considered positive for lymphomatous involvement if their short-axis diam­eter exceeded 10 mm on coronal T1- and T2-weighted STIR images. Extranodal sites included the thymus, pleura, lung, spleen, liver, kidney, bowel, bone marrow, and soft tissues. Diffusion-weighted imaging was used in order to detect potentially involved nodal and extranodal sites.
Latifoltojar et al.**^(18)^**	MRI: The disease status for the same 18 nodal disease sites and 14 extranodal disease sites. The ADC was measured by placing a region of interest in the largest cross-section of the node on the ADC map, guided by anatomically matched axial fat-suppressed T2-weighted MRI. PET/TC: Definitions of nodal disease based on long-axis size and FDG uptake in comparison with background activity.

## DISCUSSION

In this meta-analysis, we showed that WB-MRI has good accuracy for the identification of metastases in children and adolescents. This imaging modality could represent a safer option for tumor staging in pediatric patients, because it does not involve the use of ionizing radiation.

Although PET/CT is increasingly being used as the modality of choice for staging neoplasms^(19-25)^, exposure to even small doses of ionizing radiation may increase the risk of later radiation-induced malignancies, particularly in children^(26,27)^. Any PET/CT examination for staging or for the assessment of treatment response should be conducted after a clear evaluation of the risks of radiation exposure relative to the intended benefits. Considerable progress has been made in reducing the radiation dose in pediatric CT. Despite this progress and careful management, radiation exposure is still a cause for concern in pediatric patients. However, there is now evidence that the radiation dose received by children undergoing CT at adult scanning facilities can be up to twice as high as that administered at academic pediatric centers, where it is adjusted for patient size^(28)^.

The study that was excluded in the final analysis because of the high heterogeneity evaluated metastases to bone and lymph nodes^(16)^. The authors of that study reported that WB-MRI had low specificity for the detection of bone metastases. One possible explanation for this finding is the characteristic hyperintense signal of the bone marrow in the spine and pelvic bones of children and adolescents, because of the highly cellular hematopoietic bone marrow in children^(29)^. Ishigushi et al.^(16)^ obtained a number of false-positives results similar to that obtained in healthy children by Müller et al.^(29)^. However, highly cellular hematopoietic bone marrow can be correctly identified when the hyperintense signals are similar to those of the normal hematopoietic bone marrow, which is important to bear in mind when interpreting the WB-MRI findings in pediatric patients^(8,30)^. In addition, although hematopoietic bone marrow shows minimal hyperintensity in comparison with skeletal muscle, tumor infiltration produces a markedly hyperintense signal much greater than that of muscle.

Of the three MRI studies that used diffusion-weighted imaging protocols^(16-18)^, two of them^(17,18)^ also measured the apparent diffusion coefficient (ADC). One possible advantage of applying quantitative ADC cutoffs is that it can eliminate subjectivity and improve specificity in comparison with a purely visual assessment^(28)^. However, it is clear that there is an overlap in ADC values between malignant lymph nodes and normal/reactive lymph nodes, so the optimal ADC cutoff remains unclear and requires further investigation^(18)^.

We observed that the studies that utilized only STIR sequences for detecting bone metastasis^(15,28)^ achieved the best specificity and sensitivity, with no heterogeneity, and that the method had a diagnostic performance similar to that of PET/CT. Coronal STIR sequences are sensitive to soft-tissue and bone abnormalities because of their additive proton density-weighted, T1-weighted, and T2-weighted contrast with inherent fat suppression. Most pathological tissues are proton-rich and have prolonged T1 relaxation and T2 decay times, resulting in high signal intensity on fast STIR images^(28)^. Kumar et al.^(14)^ used coronal STIR parallel acquisition. Studies have also suggested that STIR WB-MRI allows excellent delineation of focal lesions that show inadequate FDG uptake, due to its high tissue contrast^(31)^. Punwani et al.^(15)^ used respiratory- and cardiac-gated axial and coronal STIR half-Fourier rapid acquisition with relaxation enhancement.

Although PET/CT had high sensitivity and specificity, the data were quite heterogeneous. One possible explanation is differences in the timing of the follow-up imaging and in the baseline neoplasms evaluated. The specificity of FDG-PET/CT was lower for the detection of bone metastases than for the detection of lymph node metastases. However, the efficiency of FDG-PET/CT for distinguishing bone metastasis from neuroblastoma was comparable to what has been reported previously^(17,32)^. In 54% of the subjects evaluated by Ishiguchi et al.^(16)^, nuclear medicine scans were taken at least 13 days after the start of treatment, including chemotherapy. The high (26.9%) false-positive rate in that study could therefore be explained by the well-known high uptake of FDG in bone marrow under chemotherapy^(33)^.

The limitations of this meta-analysis and systematic review are related to the small sample sizes in the available studies. However, the number of sites investigated was considered sufficient to support the conclusions. In addition, only one of the studies evaluated the use of WB-MRI for the detection of post-treatment disease.

In conclusion, this systematic review and meta-analysis showed that WB-MRI has good accuracy for the diagnosis of metastatic disease in children and adolescents. Because it does not expose patients to ionizing radiation, WB-MRI could represent a safer alternative for cancer staging.
